# Expression of ck-19, galectin-3 and hbme-1 in the differentiation of thyroid lesions: systematic review and diagnostic meta-analysis

**DOI:** 10.1186/1746-1596-7-97

**Published:** 2012-08-13

**Authors:** Leandro Luongo de Matos, Adriana Braz Del Giglio, Carolina Ogawa Matsubayashi, Michelle de Lima Farah, Auro Del Giglio, Maria Aparecida da Silva Pinhal

**Affiliations:** 1 Biochemistry Department – Faculdade de Medicina do ABC, Santo André, Brazil; 2 Biochemistry Department – Universidade Federal de São Paulo, São Paulo, Brazil; 3 Head and Neck Surgery Department – Faculdade de Medicina do ABC, Santo André, Brazil; 4 Medical School Students – Faculdade de Medicina do ABC, Santo André, Brazil; 5 Chairman of Hematology and Oncology Department – Faculdade de Medicina do ABC, Santo André, Brazil; Medical Oncologist – Albert Einstein Jewish Hospital, São Paulo, Brazil; 6 Rua São Paulo, 1670, Ap.41, ZIP: 09541-100, São Caetano do Sul, SP, Brazil

**Keywords:** Tumor markers, Biological, Galectin 3, Keratin 19, HBME-1 antigen, Thyroid, Review

## Abstract

**Background:**

To distinguish between malignant and benign lesions of the thyroid gland histological demonstration is often required since the fine-needle aspiration biopsy method applied pre-operatively has some limitations. In an attempt to improve diagnostic accuracy, markers using immunocytochemistry and immunohistochemistry techniques have been studied, mainly cytokeratin-19 (CK-19), galectin-3 (Gal-3) and Hector Battifora mesothelial-1 (HBME-1). However, current results remain controversial. The aim of the present article was to establish the diagnostic accuracy of CK-19, Gal-3 and HBME-1 markers, as well as their associations, in the differentiation of malignant and benign thyroid lesions.

**Methods:**

A systematic review of published articles on MEDLINE and The Cochrane Library was performed. After establishing inclusion and exclusion criteria, 66 articles were selected. The technique of meta-analysis of diagnostic accuracy was employed and global values of sensitivity, specificity, area under the summary ROC curve, and diagnostic odds ratio (dOR) were calculated.

**Results:**

For the immunohistochemistry technique, the positivity of CK-19 for the diagnosis of malignant thyroid lesions demonstrated global sensitivity of 81% and specificity of 73%; for Gal-3, sensitivity of 82% and specificity of 81%; and for HBME-1, sensitivity of 77% and specificity of 83%. The association of the three markers determined sensitivity of 85%, specificity of 97%, and diagnostic *odds ratio* of 95.1. Similar results were also found for the immunocytochemistry assay.

**Conclusion:**

This meta-analysis demonstrated that the three immunomarkers studied are accurate in pre- and postoperative diagnosis of benign and malignant thyroid lesions. Nevertheless, the search for other molecular markers must continue in order to enhance this diagnostic accuracy since the results found still show a persistency of false-negative and false-positive tests.

**Virtual slides:**

Http://www.diagnosticpathology.diagnomx.eu/vs/3436263067345159

## Introduction

Thyroid gland carcinoma is a very prevalent neoplasia worldwide. A survey sponsored by the World Health Organization (WHO) in 2010 revealed that there are around 44,670 new cases and 1,690 deaths caused by this disease every year [1].

The majority of malignant lesions of the thyroid, such as papillary carcinoma, medullary carcinoma and undifferentiated histological types, can be diagnosed by cytological criteria using samples obtained by fine-needle aspiration biopsy (FNAB) guided by ultrasonography [2]. Likewise, the diagnosis of benign lesions, such as hyperplastic nodules, colloid nodules and auto-immune diseases like thyroiditis, can be cytologically established [3]. However, to distinguish between malignant and benign lesions histological demonstration is often required for a precise diagnosis. Therefore, they are cytologically grouped as undetermined tumors or suspected follicular neoplasia [4-7] and patients often undergo a diagnostic surgical procedure (thyroidectomy) even though the general carcinoma rate of this condition is very low [8]. Thus, the immunohistochemistry method plays a complementary role in the attempt to clarify this dilemma [9].

Many studies employ immunohistochemistry techniques as an attempt to search for markers involved in the genesis or specific characteristics of follicular patterned tumors. Among the immunocytochemistry (ICC) or immunohistochemistry (IHC) markers most employed to distinguish between benign and malignant lesions of the thyroid are: cytokeratin-19 (CK-19: a keratin member family responsible for the structural integrity of epithelial cells), galectin-3 (Gal-3: involved in the process of cell migration, adherence and apoptosis) and Hector Battifora mesothelial-1 (HBME-1: an unelucidated membrane antigen that exists in the microvilli of the mesothelioma cells and also in follicular thyroid tumor cells) or their associations [10]. However, their results and applications are still controversial since these molecules have not proved to have specificity and – more critically, to avoid an eventual diagnostic thyroidectomy – enough sensitivity in the differentiation of follicular lesions because of persistent variable rates of, respectively, false-positive and false-negative results [11].

In view of this, the objective of the present study was to establish the diagnostic accuracy of CK-19, Gal-3 and HBME-1 markers, and their associations, for the differentiation between benign and malignant thyroid lesions.

## Material and methods

### Systematic review

A search for articles published exclusively in the English language between January 2001 and December 2011 was carried out in the electronic databases MEDLINE and The Cochrane Library.

A wide strategy was employed in the search in order to avoid publication bias, and the following describers were used: *((ck-19 and thyroid) OR (galectin-3 and thyroid) OR (hbme-1 and thyroid))*. Reference lists of previously obtained articles were also analyzed so that other relevant studies could be identified for inclusion in the present study.

The exclusion criteria adopted for both the study as a whole and for cases individually selected were as follows: inability to obtain individual data, review articles, case reports, use of the same sample, absence of case or control groups (as control group was considered any diagnosis of benign thyroid lesions, such as: goiter, follicular adenoma, thyroiditis, hyperplasic nodules or normal thyroid samples), fewer than 12 patients in each group (both case and control), individuals under 18 years, use of any organ other than thyroid, use of any marker other than CK-19, Gal-3 or HBME-1, inclusion of another histological malignant type other than the well-differentiated thyroid carcinoma, use of techniques other than immunocytochemistry or immunohistochemistry, use of specimens other than human, use of specimens other than those obtained exclusively from the thyroid gland (for example, blood and derivatives).

The data from the studies was independently collected by two researchers, who employed a standardized form. The following information was extracted: reference, number of patients in the case and control groups, technique employed (immunocytochemistry or immunohistochemistry), histological types of neoplasias studied and results (stratified into four groups: true-positive; false-positive; false-negative; and true-negative). Differences in the data extracted were resolved by group consensus.

**Figure 1:**
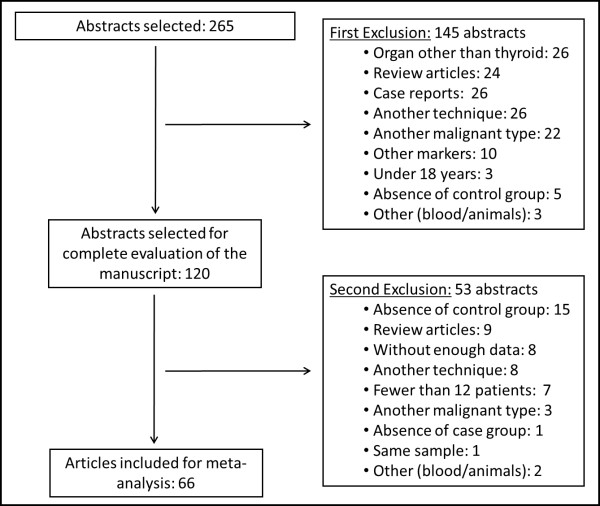
Flowchart of article selection.

Initially, 265 abstracts were selected and, after applying the established criteria above, 66 articles were included in the meta-analysis itself with 5,168 patients, as shown in Figure 1.

### Meta-analysis

The Meta-DiSc® Program (Clinical BioStatistics Unit – Hospital Ramón y Cajal, Madrid, Spain) was employed in all the analyses [12]. The method applied was the meta-analysis of diagnostic tests of independent studies stratified according to the size of the sample in each study, using Mantel-Haenszel’s method: a fixed effect estimated from the size of each study calculated by the inverse of its variance. The random effect of each study was established by the DerSimonian-Lair method and the presence of heterogeneity among the studies was estimated by the Cochrans Q-Test and was considered significant when P<0.1.

Values of sensitivity, specificity, positive and negative likelihood ratios, as well as their confidence intervals (95% CI), were calculated separately for each study and also for the studies grouped according to the type of marker or associations. Forest-plots of the most relevant results were performed.

The diagnostic *odds ratio* (dOR) was also calculated. It is an additional measure that expresses the accuracy of the test and represents how much greater the chance of achieving exactitude is when the test is positive as opposed to when the test is negative.

Complementarily, ROC (*Receiver Operating Characteristic*) analysis was done and areas under the summary ROC curves were calculated. This method is different from conventional ROC analysis, which compares test accuracy over different thresholds for positivity, because in an SROC graph each data point comes from a different study, but diagnostic thresholds should be similar for each study so as not to influence the shape of the curve [13].

## Results

The analyses of diagnostic accuracy of markers CK-19, Gal-3 and HBME-1, and their associations, in the differentiation of well-differentiated carcinoma and benign thyroid lesions, were evaluated separately by the immunohistochemistry and immunocytochemistry techniques, as described below.

### Immunohistochemistry technique

The present meta-analysis included 49 articles and 5168 patients in the broader analysis, with variable rates of true-positive and true-negative tests, and with a considerable rate of false results (Table 1).

**Table 1:** Number of studies, patients and their distributions included in each analysis by the immunohistochemistry technique

**IHC ANALYSIS**	**Studies**	**Patients**	**TP**	**FP**	**FN**	**TN**
CK-19 [10,14-33]	21	3603	1697 (47.1%)	433 (12.0%)	314 (8.7%)	1159 (32.2%)
GAL-3 [10,14-17,19,21,25-56]	39	5168	2270 (43.9%)	528 (10.2%)	408 (7.9%)	1962 (38.0%)
HBME-1 [10,14-19,21,24-27,29,30,41,51,57-61]	21	3900	1501 (38.5%)	324 (8.3%)	436 (11.2%)	1639 (42.0%)
CK-19 + HBME-1 [14,17]	2	157	84 (53.5%)	2 (1.3%)	14 (8.9%)	57 (36.3%)
GAL-3 + CK-19 [14,17]	2	164	90 (54.9%)	6 (3.7%)	9 (5.5%)	59 (36.0%)
GAL-3 + HBME-1 [14,17,46,51]	4	293	119 (40.6%)	15 (5.1%)	40 (13.7%)	119 (40.6%)
GAL-3 + HBME-1 + CK-19 [14,17,29]	3	231	121 (52.4%)	3 (1.3%)	22 (9.5%)	85 (36.8%)

LEGEND: TP (true-positive), FP (false-positive), FN (false-negative) and TN (true-negative).

The values for sensitivity, specificity, and likelihood ratios and their respective heterogeneity coefficients are detailed in Table 2 and Table 3. It was noted that these values are very discrepant and not so high when the immunomarkers are analyzed alone. Nonetheless, the association of markers can significantly increase the diagnostic rates but with an important loss of references.

**Table 2:** Sensitivity and specificity of each immunohistochemistry marker or association

**IHC ANALYSIS**	**Sensitivity (95% CI)**	**Q**	**P**	**Specificity (95% CI)**	**Q**	**P**
CK-19 [10,14-33]	0.81 (0.79-0.83)	192.02	<0.00001	0.73 (0.70-0.75)	254.49	<0.00001
GAL-3 [10,14-17,19,21,25-56]	0.82 (0.81-0.84)	341.95	<0.00001	0.81 (0.79-0.82)	512.70	<0.00001
HBME-1 [10,14-19,21,24-27,29,30,41,51,57-61]	0.77 (0.76-0.79)	298.64	<0.00001	0.83 (0.82-0.85)	444.58	<0.00001
CK-19 + HBME-1 [14,17]	0.86 (0.77-0.92)	0.41	0.5245	0.97 (0.89-1.00)	7.46	0.0063
GAL-3 + CK-19 [14,17]	0.91 (0.83-0.96)	0.01	0.9383	0.91 (0.81-0.97)	5.31	0.0212
GAL-3 + HBME-1 [14,17,46,51]	0.75 (0.67-0.81)	57.91	<0.00001	0.89 (0.82-0.94)	7.46	0.0587
GAL-3 + HBME-1 + CK-19 [14,17,29]	0.85 (0.78-0.90)	0.25	0.8826	0.97 (0.90-0.99)	7.33	0.0256

**Table 3:** Positive likelihood ratio (Positive LR) and negative likelihood ratio (Negative LR) of each immunohistochemistry marker and association

**IHC ANALYSIS**	**Positive LR (95% CI)**	**Q**	**P**	**Negative LR (95% CI)**	**Q**	**P**
CK-19 [10,14-33]	2.87 (2.10-3.92)	262.28	<0.00001	0.25 (0.18-0.35)	131.17	<0.00001
GAL-3 [10,14-17,19,21,25-56]	4.24 (3.08-5.82)	605.28	<0.00001	0.21 (0.15-0.30)	508.27	<0.00001
HBME-1 [10,14-19,21,24-27,29,30,41,51,57-61]	6.93 (4.42-10.88)	355.63	<0.00001	0.22 (0.16-0.30)	234.36	<0.00001
CK-19 + HBME-1 [14,17]	16.71 (0.38-742.57)	6.35	0.0118	0.16 (0.10-0.25)	0.11	0.7385
GAL-3 + CK-19 [14,17]	8.49 (1.04-69.17)	6.83	0.0089	0.10 (0.05-0.19)	0.15	0.6943
GAL-3 + HBME-1 [14,17,46,51]	4.92 (1.70-14.27)	13.48	0.0037	0.23 (0.03-1.70)	107.69	<0.00001
GAL-3 + HBME-1 + CK-19 [14,17,29]	17.19 (3.36-87.96)	3.69	0.1577	0.17 (0.11-0.24)	0.10	0.9508

**Figure 2:**
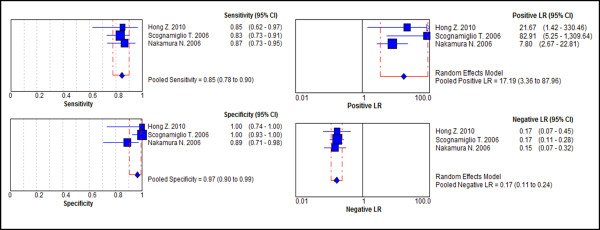
**Forest-plot graph with results for sensitivity, specificity, positive and negative likelihood ratio of immunohistochemistry expression of the positive combination of CK-19, Gal-3 and HBME-1 in the diagnosis of well-differentiated malignant thyroid lesions[**[14]**,**[17]**,**[29]**].**

Diagnostic *odds ratio* (dOR) was calculated directly from sensitivity and specificity values (Table 4). This measurement represents the overall diagnostic power of each test (a high dOR implies that the test shows good diagnostic accuracy in all patients) and, as seen, the test with greatest diagnostic accuracy and least inconsistency in the distinction between benign and malignant thyroid lesions is the positivity of the three combined markers (CK-19, Gal-3 and HBME-1). Thus, the forest-plot charts that summarize the individual results of the articles selected for this analysis in a global rate (“diamond” as pooling symbol) for sensitivity, specificity, positive and negative likelihood ratios are represented in Figure 2.

**Table 4:** Diagnostic odds ratio (dOR) calculated for each immunohistochemistry marker or combination

**IHC ANALYSIS**	**dOR (95% CI)**	**Q**	**P**
CK-19 [10,14-33]	14.73 (8.20-26.45)	110.05	<0.00001
GAL-3 [10,14-17,19,21,25-56]	23.41 (14.02-39.07)	252.72	<0.00001
HBME-1 [10,14-19,21,24-27,29,30,41,51,57-61]	40.97 (21.42-78.37)	140.82	<0.00001
CK-19 + HBME-1 [14,17]	119.06 (6.81-2080.37)	2.62	0.1058
GAL-3 + CK-19 [14,17]	86.54 (10.8-693.52)	2.89	0.8292
GAL-3 + HBME-1 [14,17,46,51]	21.94 (2.88-167.49)	21.16	0.0001
GAL-3 + HBME-1 + CK-19 [14,17,29]	95.06 (25.17-359.08)	2.23	0.3280

### Immunocytochemistry technique

The same analysis was performed for the three markers with the exclusive aim of making a preoperative diagnosis of thyroid lesions. However, the combination of markers suitable for the application of meta-analysis was not identified in the literature and the results were only based on the individual expression of each molecule.

This analysis included 17 articles with a special focus on HBME-1 analysis with 3900 samples included. False-negative and false-positive rates were significant, and diagnostic results showed that Galectin-3 had low negative LR and high sensitivity, specificity and positive LR with the highest diagnostic *odds ratio*, an analysis with less heterogeneity; demonstrating that this marker is the best at making a preoperative distinction between benign and malignant thyroid lesions. The results are described in Tables 5, 6, 7, and 8.

**Table 5:** Number of studies, patients and their distributions included in each analysis, for the immunocytochemistry technique

**ICC ANALYSIS**	**STUDIES**	**PATIENTS**	**TP**	**FP**	**FN**	**TN**
CK-19 [31,62,63]	3	230	111 (48.3%)	19 (8.3%)	27 (11.7%)	73 (31.7%)
GAL-3 [31,64-75]	14	1785	581 (33.9%)	103 (6.0%)	104 (6.0%)	927 (54.1%)
HBME-1 [31,62,64,66,74,76,77]	7	3900	1501 (38.5%)	324 (8.3%)	436 (11.2%)	1639 (42.0%)

LEGEND: TP (true-positive), FP (false-positive), FN (false-negative) and TN (true-negative).

**Table 6:** Sensitivity and specificity of each immunocytochemistry marker

**ICC ANALYSIS**	**Sensitivity (95% CI)**	**Q**	**P**	**Specificity (95% CI)**	**Q**	**P**
CK-19 [31,62,63]	0.80 (0.73-0.87)	63.04	<0.00001	0.79 (0.70-0.87)	23.50	<0.00001
GAL-3 [31,64-75]	0.85 (0.83-0.88)	64.53	<0.00001	0.90 (0.88-0.92)	48.39	<0.00001
HBME-1 [31,62,64,66,74,76,77]	0.83 (0.79-0.86)	15.30	0.0180	0.79 (0.75-0.84)	48.62	<0.00001

**Table 7:** Ratios of positive likelihood (Positive LR) and of negative likelihood (Negative LR) of each immunocytochemistry marker

**ICC ANALYSIS**	**Positive LR (95% CI)**	**Q**	**P**	**Negative LR (95% CI)**	**Q**	**P**
CK-19 [31,62,63]	(0.80-33.17)	28.16	<0.00001	0.26 (0.18-0.36)	1.48	0.4765
GAL-3 [31,64-75]	7.73 (5.54-10.79)	40.40	0.0001	0.15 (0.09-0.22)	65.52	<0.00001
HBME-1 [31,62,64,66,74,76,77]	6.71 (2.92-15.44)	52.38	<0.00001	0.20 (0.14-0.30)	15.62	0.0159

**Table 8:** Diagnostic odds ratio (dOR) calculated for each immunocytochemistry marker

**ICC ANALYSIS**	**dOR (95% CI)**	**Q**	**p**
CK-19 [31,62,63]	30.31 (12.64-72.66)	0.46	0.7954
GAL-3 [31,64-75]	64.18 (36.26-113.61)	31.98	0.0024
HBME-1 [31,62,64,66,74,76,77]	42.28 (13.02-137.28)	34.44	<0.00001

### Exploring heterogeneity

The first factor of heterogeneity loss in the analyses employing the immunohistochemistry technique was the combination of markers, as previously shown. Therefore, it became clear that none of these molecules, when studied independently, can reliably differentiate between benign and well-differentiated malignant tumors of the thyroid.

Therefore, in a search for other factors involved in the determination of heterogeneity causes, the following possible confounding variables were evaluated: inclusion of oncocytic or Hürthle cells in the sample and/or the criterion adopted to consider a marker as “positive”.

When both techniques (imunocytochemistry and immunohistochemistry) are considered, the review of the selected studies indicated that some authors actually included oncocytic patterned tumors (or Hürthle cell neoplasias) in their samples. Hürthle cells are characterized by their wide and granular cytoplasm and, besides, most oncocytic lesions at cytology are shown to be benign lesions upon histopathological examination (Hürthle cell adenomas, hyperplastic nodules, thyroiditis and Graves’ disease), the mere presence of these cells in a cytological exam indicates a greater likelihood of malignancy (Bethesda IV), regardless of other criteria [78]. These factors make an exact etiological preoperative diagnosis of these neoplasms even more difficult [79].

It was also observed that in some studies the immunostaining was considered to be positive when at least 5% of the cells expressed the marker, whereas this minimum percentage was considered by others to be 10%, 25%, or even 50%. Also, when the immunostaining was weak, heterogeneous or sometimes even focal, it was likewise, considered positive.

**Figure 3:**
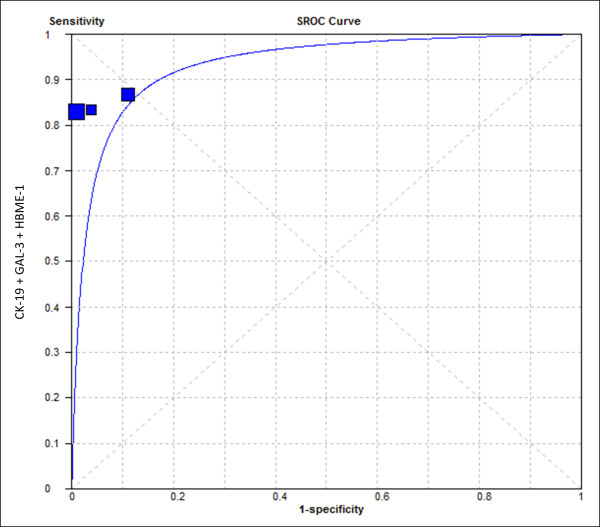
**SROC curve for positive immunohistochemistry expression of the association of the three markers (CK-19, Gal-3, HBME-1) in the diagnosis of well-differentiated malignant thyroid lesions.** Area under the SROC curve: 93.25%.

**Figure 4:**
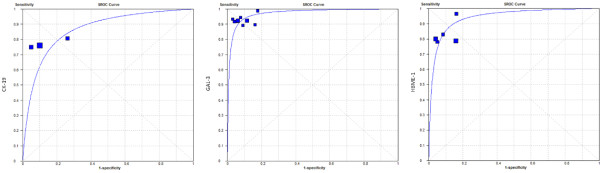
**SROC curve for positive immunocytochemistry expression of CK-19, Gal-3 and HBME-1 in the diagnosis of well-differentiated malignant lesions of the thyroid.** Areas under the SROC curve: CK-19=86.32%; Gal-3=97.07%; HBME-1=94.12%.

Thus, when the combination of markers was then analyzed (only for immunohistochemistry analysis), and after removing Hürthle cells from the data and reclassifying the cases with a percentage of immunostained cells below 25%, weak or focal marking as “negative”, it was possible to exclude the previously noted heterogeneity from the groups (data not shown). SROC curves were plotted at this time to thresholds of the different studies to make them more similar and to better illustrate these results (immunohistochemistry – Figure 3 and immunocytochemistry – Figure 4). However, when these same variables were excluded and a new analysis of CK-19, Gal-3 and HBME-1 was undertaken separately, there was always the presence of unequivocal heterogeneity for the immunohistochemical technique. As the meta-analysis was performed exclusively on published studies and did not use the authors’ original data, in some instances the criteria described above could not be applied with certainty; thus, these articles were excluded from the analysis.

## Discussions

The preoperative diagnosis of thyroid lesions is not the only challenge faced by pathologists. Very often, establishing the differential diagnosis between benignancy and malignancy of a thyroid nodule, based only on the histopathological exam, can be quite difficult.

One of the greatest research challenges involving well-differentiated thyroid carcinoma is to develop a method to enable the correct differential diagnosis between benign and malignant lesions, trying to avoid a diagnostic surgery. To really reach this objective a test would need to have an especially high sensitivity rate, [11] but it has not yet been achieved in the literature even when genomic classifiers are employed [80]. Thus, the search for a “marker” that enhances this diagnostic capability is ongoing [81].

Cytokeratin-19 (CK-19) expression in thyroid nodules is in general intense and diffuse in papillary carcinoma and heterogeneous labeling in carcinoma and in follicular adenoma, with nil or low expression in other benign lesions [30,82]. Galectins, especially galectin-3, are suggested to play a role in the pathogenesis of well-differentiated thyroid carcinoma, particularly in papillary carcinoma [83] and, therefore, it is one of the markers most commonly used to assist in distinguishing thyroid lesions. Hector Battifora mesothelial-1 (HBME-1) has been demonstrated to be important as a thyroid marker of follicular origin, with greater affinity to malignant lesions when compared to benign lesions [84]. Because of that, they are the three most used immunomarkers in pathology practice and each of them had different rates of false-negatives and false-positive results and some authors advocate that a panel of the three markers might be more helpful than the use of a single immunomarker, improving the specificity, positive and negative predictive value and thus diagnostic accuracy [85].

The main contribution of this meta-analysis was to precisely quantify the accuracy of values of these three important markers employed in clinical practice. Several literature reviews have already been published but the present study is the first to analyze cumulative data and is worthy for this reason.

As demonstrated, the association of positivity for CK-19, Gal-3 and HBME-1 in IHC assays and the preoperative expression of galectin-3 in ICC samples proved to be highly accurate tests in the distinction between benign and well-differentiated thyroid carcinoma. This is further noted when heterogeneity factors were disregarded; the SROC analysis showed a global accuracy of more than 90% in this situation.

However, these results must be analyzed with great care. Despite the fact that the accuracy rates are, in general, high there is a considerable percentage of false-results. When a diagnostic test could potentially produce a false-negative result this is not a good reason to take a watchful waiting approach, especially when a malignant neoplasm is the object of the study, and many patients are subjected to a theoretically unnecessary diagnostic surgery, with associated morbidity and mortality rates.

Another important point of this study was the determination of heterogeneity variables involved in the analysis of tumors markers employed in thyroid nodule diagnosis. Thus, the combination of markers, the exclusion of Hürthle cells and the review of what must be considered positive immunostaining were the main heterogeneity factors identified. Another possible heterogeneity factor that might be considered and that was not possible to evaluate in this research has to do with the technical methodology applied in the immunohistochemical reactions like specimen fixation, monoclonal or polyclonal antibodies, biotin-free detection method, etc. These parameters should be standardized in future works in order to achieve uniformity in the studies and improvement in diagnostic accuracy of the immunocytochemistry and immunohistochemistry methods.

Nevertheless, this study has some limitations. The present review might have been influenced by publication bias since it was limited to articles in English and included only published articles. However, the wide search criteria applied and the rigorous exclusion criteria have helped to ensure the inclusion of the most relevant studies.

In summary, this meta-analysis demonstrated that the three studied immunomarkers are accurate in making a pre- and postoperative distinction between benign and malignant thyroid lesions with accuracy of around 90% for both immunocytochemistry and immunohistochemistry assays, despite avoiding variables responsible for heterogeneity in the analysis. Although, the search for other molecular markers must continue in order to enhance this diagnostic accuracy since the results found still show persistency of false-negative and false-positive tests.

## Competing interests

The authors declare that they have no competing interests.

## Authors' contributions

ABG, COM, MLF and LLM performed the systematic review and article selection. LLM participated in the design of the study, performed the statistical analysis and wrote the manuscript. AG and MASP conceived the study, and participated in its design and coordination and helped to draft the manuscript. All authors read and approved the final manuscript.
